# Exploring Multilingual Large Language Models for Enhanced TNM Classification of Radiology Report in Lung Cancer Staging

**DOI:** 10.3390/cancers16213621

**Published:** 2024-10-26

**Authors:** Hidetoshi Matsuo, Mizuho Nishio, Takaaki Matsunaga, Koji Fujimoto, Takamichi Murakami

**Affiliations:** 1 Department of Radiology, Kobe University Graduate School of Medicine, Kobe 650-0017, Japanmurataka@med.kobe-u.ac.jp (T.M.); 2 Center for Advanced Medical Engineering Research & Development, Kobe University, Kobe 650-0047, Japan; 3 Advanced Imaging in Medical Magnetic Resonance, Kyoto University, Kyoto 606-8507, Japan; kfb@kuhp.kyoto-u.ac.jp

**Keywords:** lung cancer, multilingual large language models, radiology, TNM classification

## Abstract

Background/Objectives: This study aimed to investigate the accuracy of Tumor, Node, Metastasis (TNM) classification based on radiology reports using GPT3.5-turbo (GPT3.5) and the utility of multilingual large language models (LLMs) in both Japanese and English. Methods: Utilizing GPT3.5, we developed a system to automatically generate TNM classifications from chest computed tomography reports for lung cancer and evaluate its performance. We statistically analyzed the impact of providing full or partial TNM definitions in both languages using a generalized linear mixed model. Results: The highest accuracy was attained with full TNM definitions and radiology reports in English (M = 94%, N = 80%, T = 47%, and TNM combined = 36%). Providing definitions for each of the T, N, and M factors statistically improved their respective accuracies (T: odds ratio [OR] = 2.35, *p* < 0.001; N: OR = 1.94, *p* < 0.01; M: OR = 2.50, *p* < 0.001). Japanese reports exhibited decreased N and M accuracies (N accuracy: OR = 0.74 and M accuracy: OR = 0.21). Conclusions: This study underscores the potential of multilingual LLMs for automatic TNM classification in radiology reports. Even without additional model training, performance improvements were evident with the provided TNM definitions, indicating LLMs’ relevance in radiology contexts.

## Simple Summary

Cancer staging utilizing the TNM (Tumor, Node, Metastasis) classification system is fundamental in determining disease progression. The manual extraction and classification of TNM information from radiology reports presents significant challenges in clinical workflow efficiency and consistency. This study investigated the capability of GPT3.5, a large language model, to automate TNM classification from radiology reports in both English and Japanese languages. Our analysis demonstrated that when provided with comprehensive TNM definitions, the model achieved high accuracy rates, particularly in English reports (94% for M-stage classification). These findings indicate the potential of multilingual language models to enhance the efficiency and standardization of cancer staging processes across diverse healthcare environments.

## 1. Introduction

Radiologists compile diagnostic reports from medical images; however, the current format of these reports, which is often narrative and unstructured, limits their utility to clinicians and patients [1]. In cancer management, structuring diagnostic findings into Tumor, Node, Metastasis (TNM) classification is crucial for clinical research and prognosis [2]. Extracting TNM classifications typically demands manual effort because of the unstructured nature of radiology reports and frequent updates to classification standards, which can render previous classifications obsolete [3].

Recently, progress in deep learning in various domains has been remarkable, and its benefits have begun to be realized in real-world applications [4]. Of particular note is the development after 2017, following the introduction of the transformer, which marked a significant evolution not only in natural language but also in image processing [5,6,7,8,9,10]. The release of GPT-4 in 2023, which is known for its high performance, has demonstrated impressive results in various tasks, including the United States Medical Licensing Examination and the Radiology Board Examination [11,12]. However, observation indicates that while this model excels in addressing questions related to clinical management, its performance notably drops in classification tasks without additional training [13].

In radiology, enhancing performance in highly domain-specific tasks, where large language models (LLMs) may lack task-relevant knowledge, can be achieved by providing additional information to the LLM [14,15]. However, there have been a few investigations into the specific knowledge that should be provided [16]. Additionally, while some LLMs, like ChatGPT [17], support multiple languages, performance declines when input and output are in languages other than English, such as Japanese [12]. It has been suggested that LLM performance improves with the dataset’s size [18]. From this perspective, it seems preferable to create models supporting multiple languages rather than specializing in a single language. However, advancing this development requires evaluating performance degradation in non-English languages using multilingual LLMs.

This study employed GPT3.5-turbo (GPT3.5), a representative multilingual LLM developed by OpenAI to automatically classify TNM in radiology reports. We assessed the performance of GPT3.5 in TNM classification, considering the following: (i) the impact of providing either full text or parts of the TNM classification definition, and (ii) the language of the radiology reports.

## 2. Materials and Methods

As the evaluation of GPT3.5 utilized publicly available datasets, this study did not require ethics committee approval. We employed OpenAI’s GPT3.5 to predict TNM classifications from radiology reports of chest computed tomography (CT) scans for lung cancer in Japanese or English. We assessed the model’s performance and examined how receiving TNM definitions, or portions thereof, in both languages impacted its classification ability. Notably, we did not use CT images in this study, focusing solely on the text of the reports.

### 2.1. Dataset

**Figure 1:**
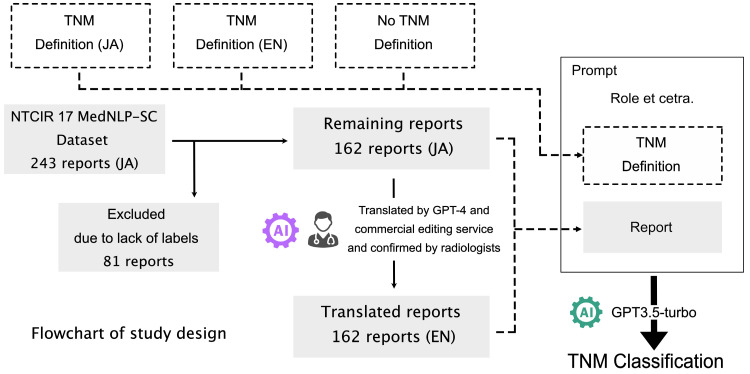
Flowchart of the study design. EN, English language; JA, Japanese language; MedNLP-SC, Medical Natural Language Processing for Social Media and Clinical Texts; NTCIR, NII Testbeds and Community for Information Access Research.

In this study, we utilized a dataset provided during the competition held in 2023 as part of the NII Testbeds and Community for Information Access Research (NTCIR-17), specifically for Medical Natural Language Processing for Social Media and Clinical Texts (MedNLP-SC) shared tasks. The dataset consisted of chest CT reports for lung cancer, documented in Japanese by board-certified radiologists, paired with their ground-truth TNM classifications. Although the dataset comprised 234 reports, only 162 radiology reports were accompanied by the ground truth, which was used to evaluate the zero-shot task (Figure 1). TNM classifications were categorized according to the 8th edition of the Union for International Cancer Control (UICC); however, within the MedNLP-SC dataset, subclassifications for each factor were eliminated, and T, N, and M were classified using integer values ranging from 0–4 to 0–3 and to 0–1, respectively [14].

The Japanese reports were first translated into English using OpenAI’s GPT-4, linguistically reviewed by native English-speaking experts, and finally reviewed by a board-certified radiologist before being used as English reports.

### 2.2. TNM Definitions

A simplified version of the UICC 8th edition definitions, excluding subclassifications, was created. When providing definitions, either the entire text or parts of the text were included in the prompts for GPT3.5. The definitions were as follows:

T factor (simplified version):

- T1: size of lung cancer, <3 cm;
- T2: size of lung cancer, 3–5 cm;
- T3: (size of lung cancer, 5–7 cm) or (local invasion of chest wall, parietal pericardium, phrenic nerve);
- T4: (size of lung cancer, >7 cm) or (invasion to the mediastinum, trachea, heart/great vessels, esophagus, vertebra, carina, recurrent laryngeal nerve).

N factor (simplified version):

- N0: no regional lymph node metastasis;
- N1: metastasis in ipsilateral peribronchial and/or hilar lymph node and intrapulmonary node;
- N2: metastasis in ipsilateral mediastinal and/or subcarinal lymph nodes;

N3: metastasis in the contralateral mediastinal, contralateral hilar, ipsilateral, contralateral scalene, or supraclavicular lymph node(s).

M factor (simplified version):

- M0: no distant metastasis;
- M1: distant metastasis.

The TNM definitions and their Japanese translations (performed by native Japanese-speaking board-certified radiologists) were decomposed into T, N, and M factors. For each factor, the presence or absence of a definition was considered, resulting in eight combinations of TNM definitions in both Japanese (JA) and English (EN). The TNM definition sentences from the eight combinations were added to the prompts (Figure 1).

### 2.3. LLM Model and Prompts

This study utilized Python with the Langchain and OpenAI packages. For the LLM, we employed OpenAI’s GPT3.5-turbo (as of 23 October 2023). Notably, GPT3.5 demonstrates favorable results in both zero-shot and few-shot learning without additional training. Therefore, we adopted a zero-shot learning approach in this study, meaning no example inputs were provided to the LLM during classification. The LLM received the following prompt (only the English version is presented for brevity). The language of the prompt was the same as that of the TNM definition. Note that <Definition of TNM> was replaced with the TNM definition, and <Report> was substituted with the report (Figure 1). The source code is available at https://github.com/gn64/src_tnm_cls_gpt (accessed on 23 October 2024).

Prompt (EN):

You are an experienced respiratory surgeon.

Based on the following TNM definitions, please generate a TNM classification for the given radiological report:

<Definition of TNM>

If there are no findings mentioned, assume that no abnormal findings were observed.

<Report>

Performance evaluation was conducted using all 162 reports, considering 32 different combinations that included the language of the TNM classification (EN and JA); whether each of the T, N, and M factors of the TNM classification was provided; and the language of the report documentation (EN and JA). Performance was assessed based on four criteria: the correct identification of T, N, and M factors individually, as well as the correct identification of the combination of T, N, and M factors (ALL) [14]. Accuracy was defined as the proportion of correct classifications for each criterion.

### 2.4. Statistical Analysis

To statistically analyze the factors contributing to T, N, M, and ALL accuracies, a generalized linear mixed model (GLMM) was employed [19]. The fixed effects included the presence or absence of T, N, and M definitions; language of the TNM definition (EN or JA); and language of the report (EN or JA), with report ID as the random effect. The outcome variable was the correctness of the TNM classification. T accuracy refers to the correct classification of the T factor, with N and M accuracy similarly referring to the correct classification of their respective factors. Statistical analyses were conducted using R (version 4.3.2) and the lme4 package (version 1.1.34). Tests for T, N, M, and ALL factors applied the Bonferroni correction, with the significance set at *p* < 0.013.

## 3. Results

### 3.1. Radiology Report Characteristics

The NTCIR17 MedNLP-SC dataset comprises 243 radiological reports. However, for this study, we required both the reports and their corresponding ground truths for TNM classification. Consequently, 81 radiology reports lacking the necessary ground truths were excluded, resulting in the utilization of 162 radiology reports from the NTCIR17 MedNLP-SC dataset for evaluation (Figure 1). Characteristics of the radiological reports in the dataset are listed in Table 1, revealing imbalanced distributions of the T, N, and M factors. For instance, among the 162 radiology reports, the distribution of T factors was as follows: T0, 3; T1, 36; T2, 54; T3, 19; and T4, 50.

### 3.2. Performance Evaluation

Table 2 presents the accuracies of the correct TNM classifications for T, N, M, and ALL factors when the full definitions of TNM were provided, categorized by the language of the TNM definition and the radiology report. It also includes the accuracies for the T, N, M, and ALL factors when TNM definitions were not provided, categorized by the language of the radiology report. Across all the combinations, the accuracies followed the order of M > N > T > ALL.

**Figure 2:**
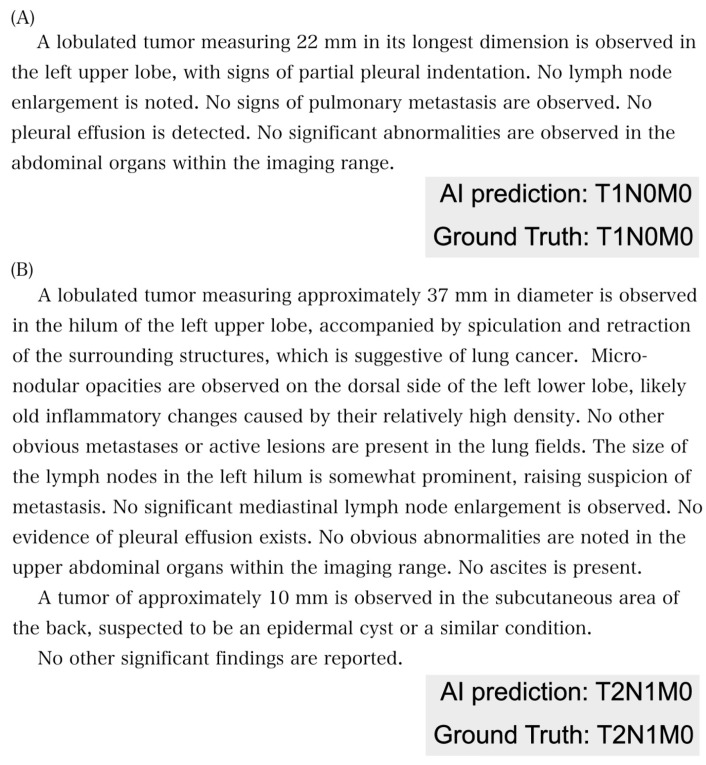
(**A**,**B**). Examples of a report correctly answered by GPT3.5-turbo.

**Figure 3:**
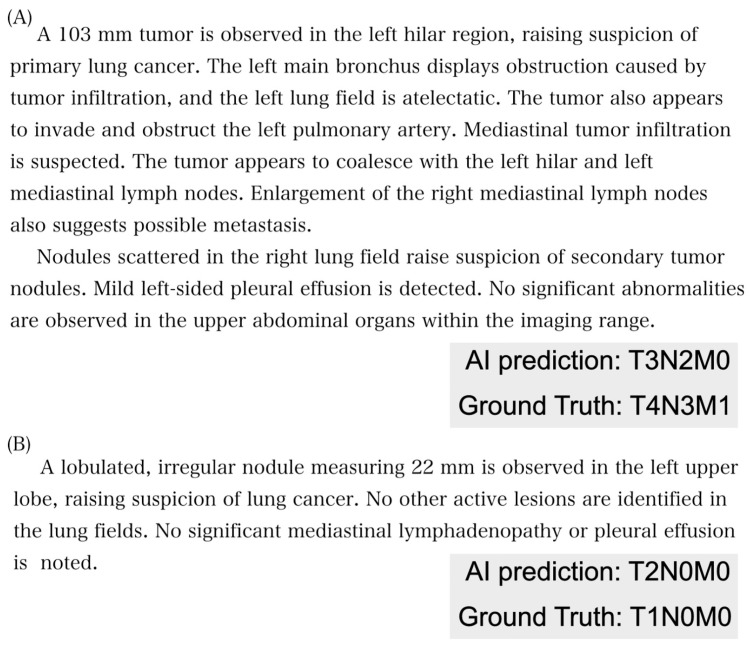
(**A**,**B**). Examples of an incorrect answer by GPT3.5-turbo.

The accuracy of the TNM classification varied depending on the presence of TNM definitions and the language used. Overall, providing TNM definitions led to better performance compared to when definitions were not provided (with full TNM definition: T accuracy, 0.44–0.57; N accuracy, 0.72–0.80; M accuracy, 0.85–0.95; ALL accuracy, 0.30–0.43; without TNM definition: T accuracy, 0.29–0.31; N accuracy, 0.57–0.70; M accuracy, 0.72–0.86; ALL accuracy, 0.20–0.23). The accuracy for each factor corresponded with the expected difficulty based on the number of available choices. The M factor classification consistently showed the highest accuracy (up to 0.94 when both the definition and report were in English), while the T factor classification proved to be the most challenging (as low as 0.29 without definitions in English reports). Representative examples of radiology reports, correctly and incorrectly predicted using GPT3.5, are shown in Figure 2 and Figure 3, respectively. Correct predictions are evident in Figure 2A (T1N0M0) and Figure 2B (T2N1M0), while GPT3.5 incorrectly predicted all TNM factors in Figure 3A and inaccurately predicted the T factor in Figure 3B.

### 3.3. Statistical Analysis Using GLMM

Table 3 and Table 4 present the results of the statistical analysis conducted using the GLMM to identify factors contributing to the accuracy of T, N, M, and ALL. The odds ratios (OR) and *p*-values for each explanatory variable are presented in Table 3 and Table 4. For the T accuracy, the definition of T emerged as a significant explanatory variable that enhanced performance (OR = 2.35 95% CI 2.06–2.69), whereas the definitions of N and M, languages of radiology reports, and TNM definitions were not significant explanatory variables. For the N accuracy, significant improvements were observed with the definitions of N and M (OR = 1.94 95% CI 1.68–2.25 and 1.27 95% CI 1.10–1.46, respectively), and a significant decrease in performance was observed when the reports were documented in Japanese (OR = 0.74 95% CI 0.64–0.85). Regarding the M accuracy, significant improvements were evident with the T and M definitions (OR = 1.52 95% CI 1.25–1.85 and 2.50 95% CI 2.04–3.06, respectively), particularly with the M definition. Notably, documenting reports in Japanese led to a significant decrease in performance (OR = 0.21 95% CI 0.17–0.27) for the M accuracy. For the overall TNM classification (ALL accuracy), the definitions of T, N, and M showed significantly improved performance (OR = 1.44 95% CI 1.24–1.68, 1.56 95% CI 1.34–1.82, and 1.28 95% CI 1.10–1.49, respectively).

## 4. Discussion

This study demonstrated that multilingual LLMs (GPT3.5) can achieve promising performance in classifying TNM from radiology reports; however, further improvements are necessary before clinical implementation. Overall, providing TNM definitions enhances LLMs’ performance in TNM classification. The improvement in LLMs performance for the T-factor is strongly influenced by the definition of T (OR 2.35), for the N-factor by the definitions of N and M (OR 1.94 and 1.27), and for the M-factor by the definitions of T and M (OR 1.52, 2.50). Consistently, definitions pertaining to the same factors lead to significant improvements. While Japanese language in radiology reports significantly decreased N and M accuracies, T and ALL accuracies were not significantly affected by language. The degradation in performance due to the use of Japanese in some parts is considered to reflect the volume of the training dataset of the LLM; however, as seen in T and ALL accuracies, there are areas where the impact of language is minimal, and it cannot be categorically stated that the use of Japanese leads to decreased performance.

Previous studies reported that ChatGPT struggles, particularly in classification and calculation tasks [13]. However, the results of the current study have demonstrated that providing definitions can significantly improve the accuracy of classification tasks of TNM definitions from radiology reports.

GPT3.5 demonstrated a certain level of performance even without TNM definitions, suggesting innate knowledge of TNM classification. However, as demonstrated in Table 2, supplying TNM definitions significantly enhanced GPT3.5’s performance, suggesting the feasibility of imparting knowledge and definitions of TNM classification via prompts. This approach holds promise for other highly domain-specific tasks, such as TNM classification.

The LLMs performance can deteriorate when inputting and outputting languages other than English, such as Japanese [12]. When assessing the impact of language on T, N, M, and ALL accuracy, a significant difference was observed in using Japanese reports for predicting N and M factors (OR = 0.74 and 0.21). In addition, there was no strong performance deterioration attributed to language in TNM definitions. Given that the radiology reports in the NTCIR17 MedNLP-SC dataset were originally recorded in Japanese, it is speculated that the performance deterioration in this study could be linked to GPT3.5’s proficiency in Japanese.

Regardless of whether TNM definitions were provided, accuracies followed the order of M > N > T > ALL, which, in conjunction with the number of available options for each factor (five for T, four for N, and two for M), suggested that T was the most challenging, followed by N and M. The definition of T contributed to the performance improvement not only in T accuracy but also in N and M accuracies, which could be partly attributed to the correlation between the characteristics of the primary tumor (T) and the patterns of its metastasis (N and M). In the present study, the definition of M contributed strongly to the accuracy of M, despite its low level of difficulty. M1 was classified into three substages (M1a, M1b, and M1c) in the 8th edition of the UICC, but the M factor was simplified into two categories (M0 and M1) in this dataset. This simplification differs from the general definition of the TNM. This may be the reason for the significant improvement in the M accuracy.

Various studies have been conducted concerning the use of LLMs in the generation of radiology reports [20,21]. According to the findings reported by Sun et al., when chest X-ray reports generated by GPT-4 were compared with those written by radiologists, the latter were deemed to have higher coherence, comprehensiveness, and factual consistency, as well as presenting less medical harm from the radiologists’ viewpoint. Conversely, referring physicians have found LLM-generated reports to be more favorable [20,21]. This study can be considered an exploration of the use of LLMs to enhance the value of radiologists’ reports without increasing their workload. Recently, specialized LLMs for radiology have emerged [22,23], and the overall performance improvements of general LLMs can provide significant support to radiologists and physicians.

This study had several limitations. First, the number of radiology reports (N = 162) used for the performance evaluation was small. However, this study uses zero-shot learning, which mitigates concerns regarding overfitting. Second, to observe performance fluctuations due to the English and Japanese texts, we used translated texts. While the translated texts were reviewed and corrected by specialists whose native language was Japanese or English, the use of translated texts may affect the model’s performance. Third, this study utilized the NTCIR-17 dataset, and the TNM classifications for the reports were based on ground-truth classified by board-certified radiologists in NTCIR-17. Consequently, the evaluation in this study is dependent on the quality of this ground-truth. Fourth, this study considered multiple combinations and utilized GPT3.5, which was the available model from OpenAI at the time of study; however, it may not be the most advanced model currently available. Finally, this study relied on the general LLM, GPT3.5, to assess performance, and it is essential to also consider radiology-specific LLMs, which have been actively researched recently.

## 5. Conclusions

This study demonstrates the potential of multilingual large language models (LLMs) in automating TNM classification from radiological reports for lung cancer. Using GPT-3.5-turbo, we have shown that these models can achieve significant improvements in accuracy when classifying tumor (T), node (N), and metastasis (M) stages, particularly when comprehensive TNM definitions are provided.

Our findings reveal that the highest accuracy was achieved with English reports and complete TNM definitions, highlighting the importance of clear, standardized input in optimizing model performance. Providing specific definitions for T, N, and M factors resulted in substantial improvements in their respective classification accuracies, emphasizing the value of context-rich prompts in enhancing LLM output. While overall performance was strong, there were variations in accuracy across T, N, and M classifications, with the M classification showing the highest accuracy and the T classification the lowest. This variability suggests room for future refinement and targeted improvements in the classification process.

The observation of decreased accuracy for N and M classifications in Japanese reports points to potential language-specific challenges that warrant further investigation and adaptation of the model or prompting strategies. The ability to automatically generate accurate TNM classifications from radiology reports has the potential to significantly streamline workflow, reduce human error, and enhance consistency in cancer staging.

## Figures and Tables

**Table 1:** Radiology Report Characteristics, including T, N, and M factors.

Characteristics	Total	Proportion
T factor		
0	3	0.02
1	36	0.22
2	54	0.33
3	19	0.12
4	50	0.31
N factor		
0	66	0.41
1	18	0.11
2	62	0.38
3	16	0.10
M factor		
0	101	0.62
1	61	0.38

M, metastasis; N, node; T, tumor.

**Table 2:** T, N, M, and ALL accuracies with and without the full TNM definitions.

With Full TNM Definition					
TNM definition	Report	T accuracy	N accuracy	M accuracy	ALL accuracy
EN	EN	0.47 (76/162)	0.80 (131/162)	0.94 (153/162)	0.36 (58/162)
EN	JA	0.57 (92/162)	0.75 (121/162)	0.85 (138/162)	0.39 (63/162)
JA	EN	0.44 (71/162)	0.72 (116/162)	0.95 (154/162)	0.30 (48/162)
JA	JA	0.57 (93/162)	0.80 (129/162)	0.87 (141/162)	0.43 (69/162)
**Without TNM Definition**					
TNM definition	Report	T accuracy	N accuracy	M accuracy	ALL accuracy
-	EN	0.29 (47/162)	0.70 (114/162)	0.86 (139/162)	0.20 (33/162)
-	JA	0.31 (50/162)	0.57 (93/162)	0.72 (117/162)	0.23 (37/162)

ALL, the combination of the TNM factors; EN, English language; JA, Japanese language; M, metastasis; N, node; T, tumor.

**Table 3:** Statistical analysis of explanatory variables for T, N, and M accuracies using GLMM.

Object Variable	T Accuracy	
Explanatory Variable	Multivariable Model OR (95%CI)	*p*-Value
TNM definition		
Without T definition	reference	reference
With T definition	2.35 (95% CI 2.06–2.69)	<0.001
Without N definition	reference	reference
With N definition	1.16 (95% CI 1.02–1.32)	0.02
Without M definition	reference	reference
With M definition	1.12 (95% CI 0.98–1.27)	0.09
Language		
TNM in EN	1.00 (reference)	reference
TNM in JA	1.03 (95% CI 0.91–1.17)	0.64
Report in EN	1.00 (reference)	reference
Report in JA	1.12 (95% CI 0.98–1.27)	0.09
**Object Variable**	**N Accuracy**	
**Explanatory Variable**	**Multivariable Model OR (95%CI)**	***p*-Value**
TNM definition		
Without T definition	reference	reference
With T definition	0.97 (95% CI 0.84–1.12)	0.71
Without N definition	reference	reference
With N definition	1.94 (95% CI 1.68–2.25)	<0.001
Without M definition	reference	reference
With M definition	1.27 (95% CI 1.10–1.46)	0.001
Language		
TNM in EN	1.00 (reference)	reference
TNM in JA	0.92 (95% CI 0.80–1.07)	0.27
Report in EN	1.00 (reference)	reference
Report in JA	0.74 (95% CI 0.64–0.85)	<0.001
**Object Variable**	**M Accuracy**	
**Explanatory Variable**	**Multivariable Model OR (95%CI)**	***p*-Value**
TNM definition		
Without T definition	reference	reference
With T definition	1.52 (95% CI 1.25–1.85)	<0.001
Without N definition	reference	reference
With N definition	1.18 (95% CI 0.97–1.43)	0.10
Without M definition	reference	reference
With M definition	2.50 (95% CI 2.04–3.06)	<0.001
Language		
TNM in EN	1.00 (reference)	reference
TNM in JA	1.12 (95% CI 0.92–1.36)	0.25
Report in EN	1.00 (reference)	reference
Report in JA	0.21 (95% CI 0.17–0.27)	<0.001

CI, confidence interval; EN, English language; GLMM, generalized linear mixed model; JA, Japanese language; M, metastasis; N, node; OR, odds ratio; T, tumor.

**Table 4:** Statistical analysis of explanatory variables for the ALL accuracy using GLMM.

Object Variable	ALL Accuracy	
Explanatory Variable	Multivariable Model OR (95%CI)	*p*-Value
TNM definition		
Without T definition	reference	reference
With T definition	1.44 (95% CI 1.24–1.68)	<0.001
Without N definition	reference	reference
With N definition	1.56 (95% CI 1.34–1.82)	<0.001
Without T definition	reference	reference
With M definition	1.28 (95% CI 1.10–1.49)	0.001
Language		
TNM in EN	1.00 (reference)	reference
TNM in JA	0.94 (95% CI 0.81–1.09)	0.42
Report in EN	1.00 (reference)	reference
Report in JA	1.11 (95% CI 0.95–1.29)	0.18

ALL, the combination of the TNM factors; EN, English language; GLMM, generalized linear mixed model; JA, Japanese language; M, metastasis; N, node; T, tumor.

## Data Availability

Restrictions apply to the availability of these data. Data were obtained from NTCIR-17 and are available https://research.nii.ac.jp/ntcir/ntcir-17/ with the permission of NTCIR Project.
